# Significantly enhanced coupling effect and gap plasmon resonance in a MIM-cavity based sensing structure

**DOI:** 10.1038/s41598-021-98001-z

**Published:** 2021-09-16

**Authors:** Yuan-Fong Chou Chau, Tan Yu Ming, Chung-Ting Chou Chao, Roshan Thotagamuge, Muhammad Raziq Rahimi Kooh, Hung Ji Huang, Chee Ming Lim, Hai-Pang Chiang

**Affiliations:** 1 Centre for Advanced Material and Energy Sciences, Universiti Brunei Darussalam, Tungku Link, Gadong, BE1410 Brunei; 2 Department of Optoelectronics and Materials Technology, National Taiwan Ocean University, Keelung, 20224 Taiwan; 3 Taiwan Instrument Research Institute, National Applied Research Laboratories, Hsinchu, 300 Taiwan

**Keywords:** Nanoscience and technology, Nanoscale devices

## Abstract

Herein, we design a high sensitivity with a multi-mode plasmonic sensor based on the square ring-shaped resonators containing silver nanorods together with a metal–insulator-metal bus waveguide. The finite element method can analyze the structure's transmittance properties and electromagnetic field distributions in detail. Results show that the coupling effect between the bus waveguide and the side-coupled resonator can enhance by generating gap plasmon resonance among the silver nanorods, increasing the cavity plasmon mode in the resonator. The suggested structure obtained a relatively high sensitivity and acceptable figure of merit and quality factor of about 2473 nm/RIU (refractive index unit), 34.18 1/RIU, and 56.35, respectively. Thus, the plasmonic sensor is ideal for lab-on-chip in gas and biochemical analysis and can significantly enhance the sensitivity by 177% compared to the regular one. Furthermore, the designed structure can apply in nanophotonic devices, and the range of the detected refractive index is suitable for gases and fluids (e.g., gas, isopropanol, optical oil, and glucose solution).

## Introduction

Surface plasmon polaritons (SPPs) are surface electromagnetic (EM) wave modes arising from the coupling of free electrons and incident photons on the surface of metal-dielectric boundary to improve the collective vibration of electrons^1–7^. SPPs wave rises above the light diffraction limit and can confine the light within nanoscale; consequently, they have wide-ranging applications of SPPs wave in photonic integration circuits^8–12^. Optical devises depending on SPP waveguides, e.g., filters^13,14^, modulators^15,16^, absorbers^17,18^, demultiplexer^19^, amplifiers^20,21^, switches^22,23^, and sensors^24–28^ have been investigated and designed. Among them, metal–insulator-metal (MIM) waveguides with long propagation distance, low loss, strong light confinement, inexpensive production, and ease of manufacture and integration have received considerable interest and attention^26,29–31^.

Plasmonic sensors based on MIM-cavity waveguide configuration are commonly used for refractive index sensing since their feedback feature is a small variation in the surrounding material^32,33^. Many MIM-cavity designs based on SPPs to improve the sensing performance have been proposed^27,34–37^. The optimized cavity (or resonator) design can offer the best sensing performance. The device performance is associated with the cavity profile since the light-matter interaction between the bus waveguide and the resonator. Side-coupled or direct-coupled cavities with different aspects suffer an important role in producing a better light-matter interaction in a MIM-cavity waveguide system^38,39^. The multiple resonance modes receive a wide range of operation wavelengths for designing flexibly adjustable integrated optical circuits (IOCs). Multiple resonance modes can broadly adopt in multi-channel biosensors, enhancing multiband second-harmonic generation, multiband slow-light devices, and multi-wavelength surface-enhanced spectroscopy^40^. In the past few years, different MIM-cavity patterns of the plasmonic sensors such as rectangular cavities^41,42^, nanodisk cavities^43–45^, metallic double-baffle^25^, crossed ring-shaped metasurface^46^, gear-shaped nanocavity^28^, T-shaped resonators^47–49^, tooth-shaped cavities^50^, semi-ring cavity^24,51^ and racetrack ring resonator^52^, have been proposed. Specifically, certain designs used the nanoscale coupled gap resonators^53–56^ to design the plasmonic refractive index sensors based on the gap plasmon resonance (GPR) effect, which can significantly enhance the resonator's SPPs mode. P. Albella and co-workers proposed a tunable grating-based plasmonics sensor showing high sensitivity and controlled unidirectionality^56^. As reported in^55^, a silver nanorod array embedded into a square resonator was proposed and can apply for blood group identification. Also, M. R. Rakhshani et al. introduced a nanorod array coupled with two slot cavities to detect glucose concentration in water^53^. In^57^, the authors designed a metal substrate with two MIM waveguides coupled with an array of hexagonal nanoholes for hemoglobin and DNA quantification. A plasmonic MIM-cavity waveguide consisting of one rectangular cavity and three silver baffles was investigated in our previous work^58^, acting as a plasmonic sensor with a filter function. The introduction of metal nanorods, nanoholes, or baffles inside the resonator can remarkably improve the sensing performance due to their excellent optical properties in the EM field confinement and low propagation losses. However, it is difficult to implement independent tunability for multiple resonance modes arising from the collective manner in the plasmonic MIM-cavity waveguide system.Besides, the resonance modes found in the resonator greatly impact the coupling efficiency between the bus waveguide and resonance cavity, which is less discussed before and needs to be further studied.

This paper scrutinized a multiband plasmonic sensor based on a side-coupled resonator in a MIM-cavity waveguide with the refractive index sensing capability in the near-infrared wavelength range. We investigated three types of side-coupled cavity configurations, i.e., one square ring, one square ring with silver nanorods, and double square ring with silver nanorods, respectively. Simulations are employed using the two-dimensional (2-D) finite element method (FEM) for resonance mode analysis and sensing performance characterization. We found that the structure with double square rings, including silver nanorods, can significantly increase the coupling effect between the bus waveguide and the side-coupled resonator since the resonators enhanced the GPR modes. Furthermore, the suggested structure improves the sensing performance because of the excellent interaction with the surrounding medium under detecting. Generally, our plasmonic sensor can promote sensitivity through the phenomenon of plasmon exciting among the gap surface of the nanorods and would be beneficial to the application in the biomedical field and the implementation in IOCs. In this work, we show its applicability for the detection of different fluids as well.

## Simulation model and analysis method

**Figure 1:**
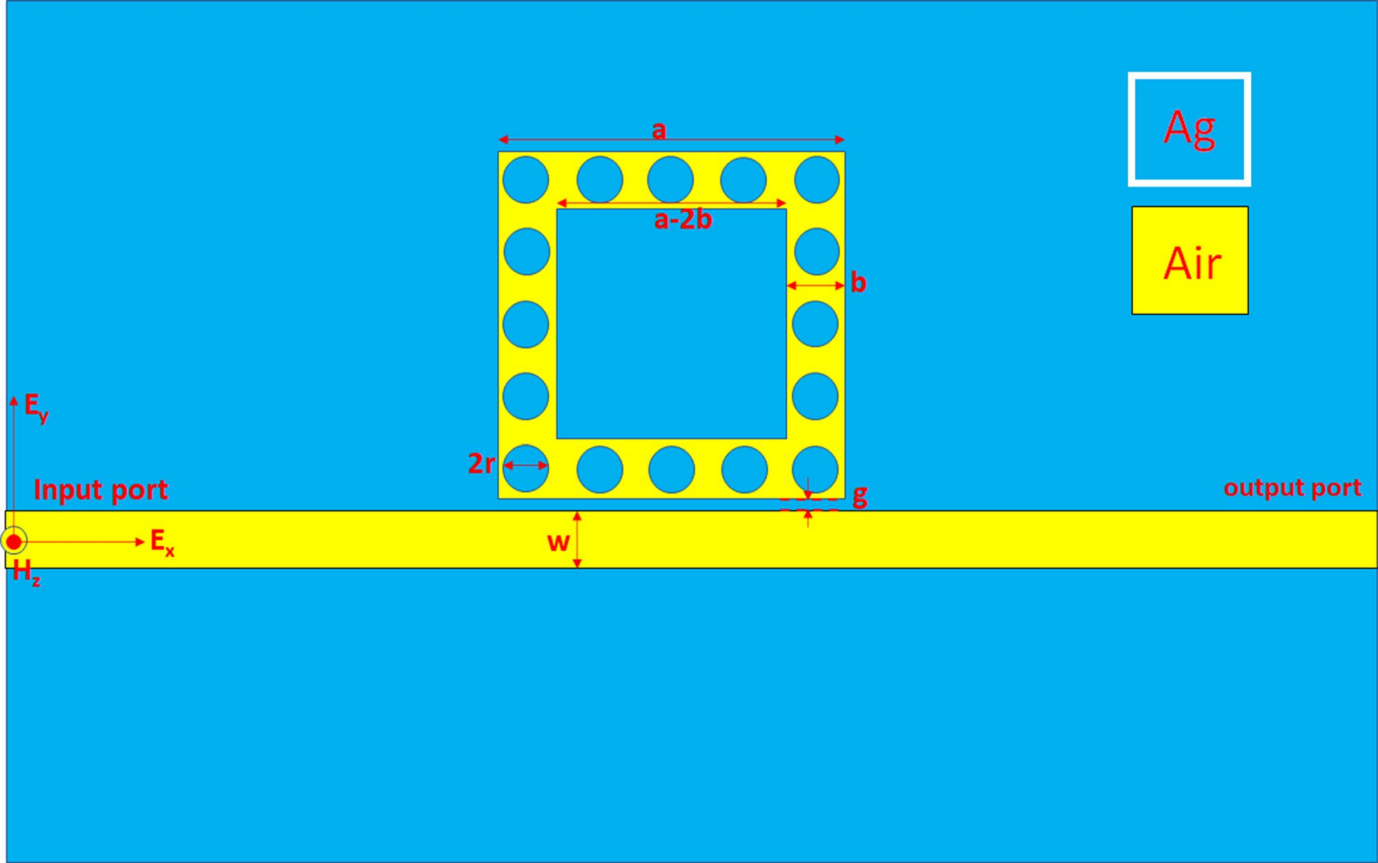
Top view of the investigated plasmonic sensor, consisting of a MIM bus waveguide coupled with one square ring-shaped cavity containing sixteen nanorods (with radius *r*) uniformly distributed in the resonance ring.

Figure 1 displays the top view of the investigated plasmonic sensor, consisting of a MIM bus waveguide coupled with one square ring-shaped cavity containing sixteen nanorods (with radius *r*) uniformly distributed in the resonance ring. We indicated the structural parameters in Fig. 1,i.e., the gap distance between the bus waveguide and the square ring-shaped cavity is *g*, the outer and inner lengths of the square resonator are *a* and *a-2w*, respectively. In Fig. 1, the cyan- and yellow-colored regions stand for the silver and insulator medium (air with the refractive index of *n* = 1.00), respectively. The investigated structure in the *z*-direction is infinite in the simulations, and this simulation system is a 2-D model. We employed a commercially available FEM (COMSOL Multiphysics^59,60^) with perfectly matched layer (PML) absorbing boundary conditions for soaking up the outgoing light. As a result, the investigated structure's subdomains can partition into triangular mesh elements with an “ultra-fine” mesh grid size. This setting permits us to obtain precise simulation results within the available computer resources. A TM-polarized EM wave coupled with the fundamental SPP mode^61–63^ into the bus waveguide's input port^64^. In Fig. 1, H_z_ is the magnetic field component in the z-direction, E_x_ is the electric field component in the x-direction, and E_y_ is the electric field component in the y-direction.

Silver (Ag) was chosen as the plasmonic material to generate an EM wave response within the near-infrared range since its small imaginary part of the relative permittivity and lower power consumption. The relative permittivity (ε_m_) of silver can characterize by the Drude model^65^.

1 $${\upvarepsilon }_{\mathrm{m}}\left(\upomega \right)={\upvarepsilon }_{\infty }-\frac{{\upomega }_{\mathrm{p}}^{2}}{{\omega }^{2}+i\omega \gamma }$$

where ε_∞_ = 3.7 (the infinite dielectric constant), ω is the frequency of incident EM wave, ω_p_ = 9.10 eV (bulk plasma frequency), and γ = 18 meV (the electron collision frequency).

The input and output ports are located at the left and right sides of the designed structure with the same length from the center of the bus waveguide to detect the input and output powers. The transmittance (T) can obtain by T = P_out_ (output power)/P_in_ (input power), where the P_out_ and P_in_ can calculate as integral values of energy-flux density. The square ring can act as a Fabry–Pérot cavity, and the resonance will happen when the SPPs are coupled into the ring resonator and fit the resonance condition. For a MIM waveguide-cavity system, the SPPs can be excited when the incident EM wave approaches the intrinsic resonance wavelength (λ_res_). If Δφ = 2π*N* (*N* is an integer), the λ_res_ can be expressed by temporal coupled-mode theory^66,67^.

2 $${{\uplambda }_{\mathrm{res}}=\frac{2{L}_{eff}{Re(n}_{\mathrm{eff}})}{N-\frac{\varphi }{\pi }} (N=\mathrm{1,2},3\dots )}$$

Here, *N* denotes the order of the standing wave resonance, *L*_eff_ represents the effective length of the cavity, φ stands for the phase shift, and Re(*n*_eff_) is the real part of the effective refractive index. *n*_eff_ can describe as:

3 $${Re(n}_{\mathrm{eff}})={\left({\varepsilon }_{Ag}+{\left(\frac{k}{{k}_{0}}\right)}^{2}\right)}^{1/2}$$

where *k* = 2π/λ is the wave vector in the waveguide and *k*_0_ is the wave vector in the free space.

The definition of sensitivity (S) is

4 $$\mathrm{S}=\frac{\mathrm{\Delta \lambda }({\text{the shift of }}{\uplambda }_{res})}{\Delta n ({\text{the change in the refractive index}})}({\text{nanometer per refractive index unit}},\mathrm{ nm}/\mathrm{RIU})$$

The figure of merit (FOM) and quality factor (Q factor) are S/FWHM and λ_res_ /FWHM, respectively, where FWHM is the full width at half-maximum of the λ_res_.

Since the fast progress in nanophotonics, the manufacturing of the investigated structure is attainable with current fabrication technologies, allowing cost-effective fabrication over a large region^68^. The MIM waveguide with a rectangular ring can realize by using stripping and ion beam lithography processes^69^. The Ag nanorods can be made with high aspect ratios by focus ion beam etching^68^. However, the object of this paper is not to concentrate on the fabrication procedures. As an alternative, several potential articles that investigated in-depth of this topic are advised^70–72^.

## Results

**Figure 2:**
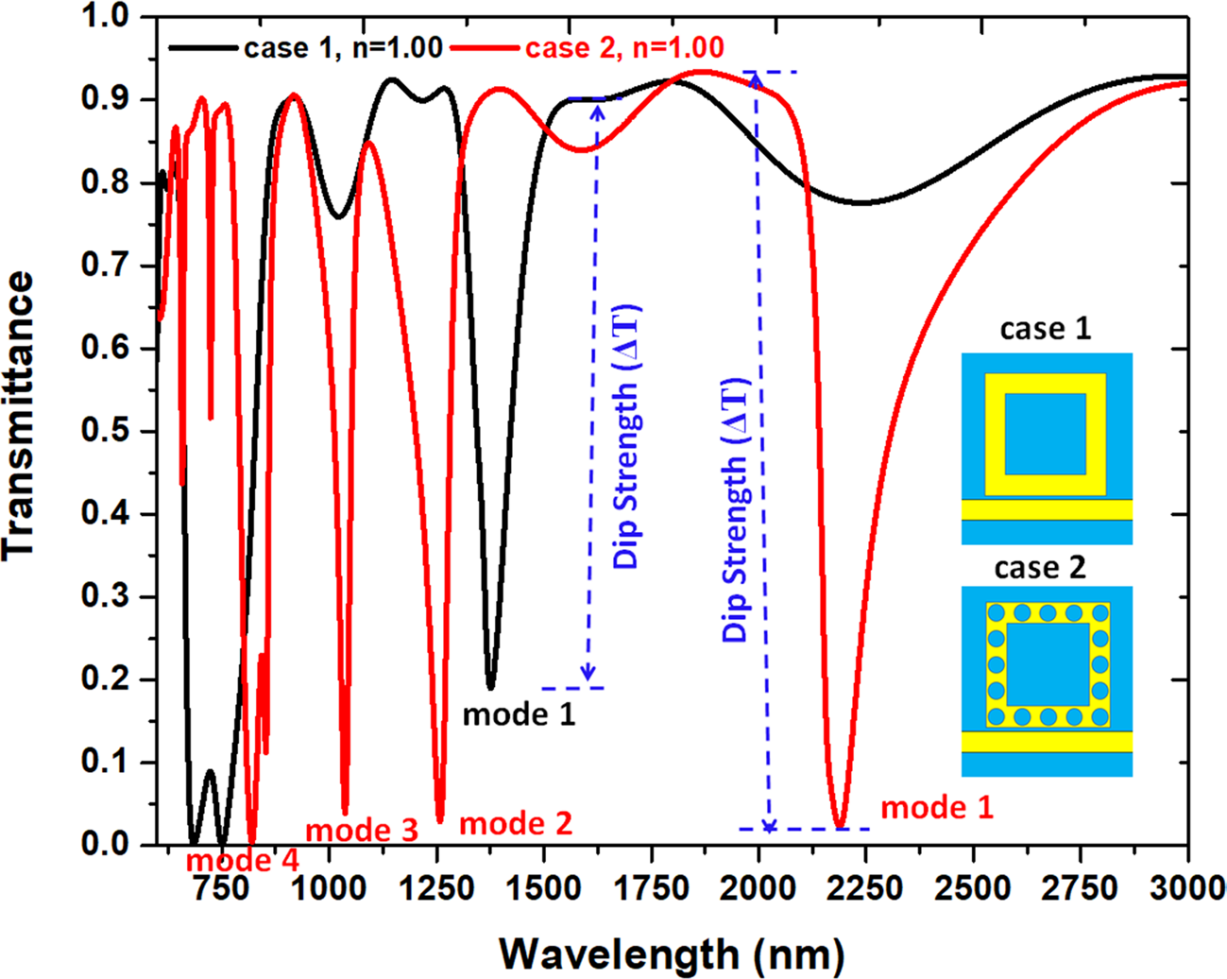
Comparison of the transmittance spectrum of the SPPs modes of two waveguide-cavity types, i.e., a bus waveguide coupled to one square air ring (black, denoted as case 1), and a bus waveguide coupled to one square air ring containing sixteen nanorods (with radius *r*) uniformly distributed in an air ring (red, denoted as case 2). The structural parameters w, *a*, *g*, *b*, and *r* are 50 nm, 300 nm, 10 nm, 50 nm, and 20 nm, respectively.

Figure 2 compares the transmittance spectrum of the SPPs mode for two waveguide-cavity types, i.e., a bus waveguide coupled to one square air ring (black, denoted as case 1), and a bus waveguide coupled to one square air ring containing sixteen nanorods (with radius *r*) uniformly distributed in an air ring (red, denoted as case 2). To ensure only the TM mode can travel in the designed structure, the bus waveguide and square ring’s widths are *w* = 50 nm throughout this paper, otherwise specified. The default structural parameters, *a*, *g*, *b*, and *r*, signify as 300 nm, 10 nm, 50 nm, and 20 nm, respectively. Besides, the difference between the maximum and minimum transmittance is the dip strength (ΔT)^73^. As shown, an apparent discrepancy of the optical spectrum concerning the different resonance modes can clarify this dissimilarity after the silver nanorods exist in the investigated plasmonic waveguide-cavity system. In case 1, only one available transmittance dip corresponding to an SPP mode at λ_res_ = 1376 nm can be observed. This SPP mode is due to the surface plasmon resonance (SPR) and cavity plasmon resonance (CPR) from the coupling effect between the square air ring and the MIM bus waveguide. When the silver nanorods appear in the square air ring, case 2 can generate more SPPs modes because of the enhanced SPR, CPR and GPR effects among the silver nanorods, leading to four available SPPs modes at λ_res_ = 2188 nm, 1258 nm, 1037 nm, and 820 nm, respectively. The interference of surface, cavity, and gap plasmon resonances cause the multiple SPPs modes among bus waveguides, square air rings, and silver nanorods^74–79^. We found that the GPR effect plays a pivotal role in offering more plasmon resonance in the investigated waveguide-cavity system. The resonance dip in case 2 has a muscular dip strength and a narrow FWHM, both beneficial to refractive index sensing performance. This remarkable feature has led to possible applications in IOCs.

**Figure 3:**
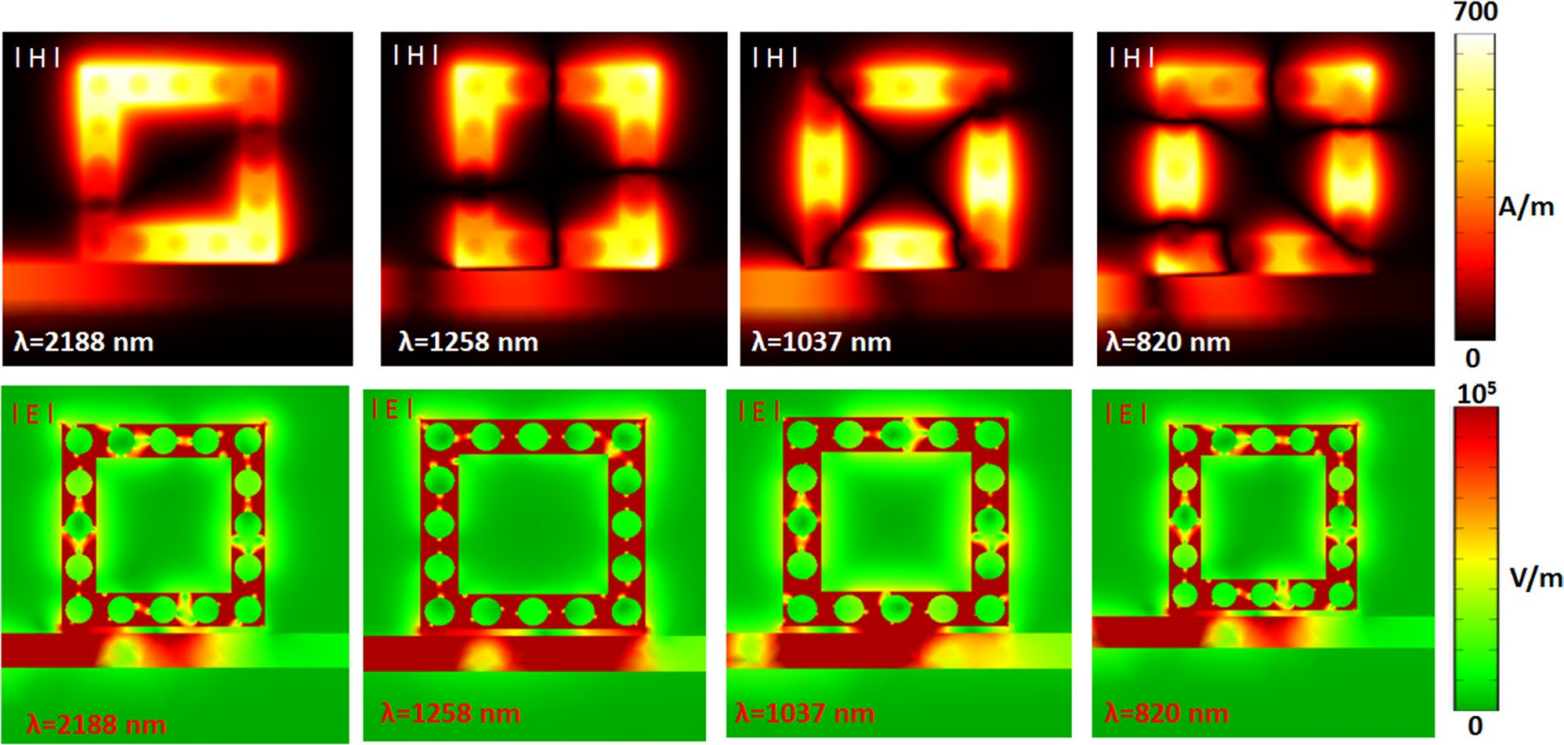
Truncate views of magnetic field (|H|) and electric field (|E|) patterns at the corresponding wavelengths from mode 1 to mode 4 in case 2 structure, respectively.

To understand the physical nature, Fig. 3 shows the steady-state of the magnetic field (|H|) and electric field (|E|) patterns at corresponding λ_res_ from modes 1 to 4. As seen, the standing wave occurs in the square air ring resonator at λ_res_, and most input EM wave confines in the resonance cavity. The incident wavelength highly influences the |H| patterns of SPPs modes due to the different optical waves^80^. The light spot number of |H| patterns in square air rings are 2, 4, 4, and 6 when the λ_res_ is varied from 2188 nm, 1258 nm, 1037 nm, and 820 nm, respectively. Thus, the square air ring can behave as a Fabry-Pérot cavity in the investigated plasmonic sensor system. According to the |E| field patterns, the SPPs wave can couple well because of the constructive interference between the bus waveguide and the ring resonator, showing significant GPR among the silver nanorods. The |H| and |E| field enhancement of the SPPs modes in the gap space of silver nanorods exhibit an excellent light-matter coupling in the square ring resonator.

**Figure 4:**
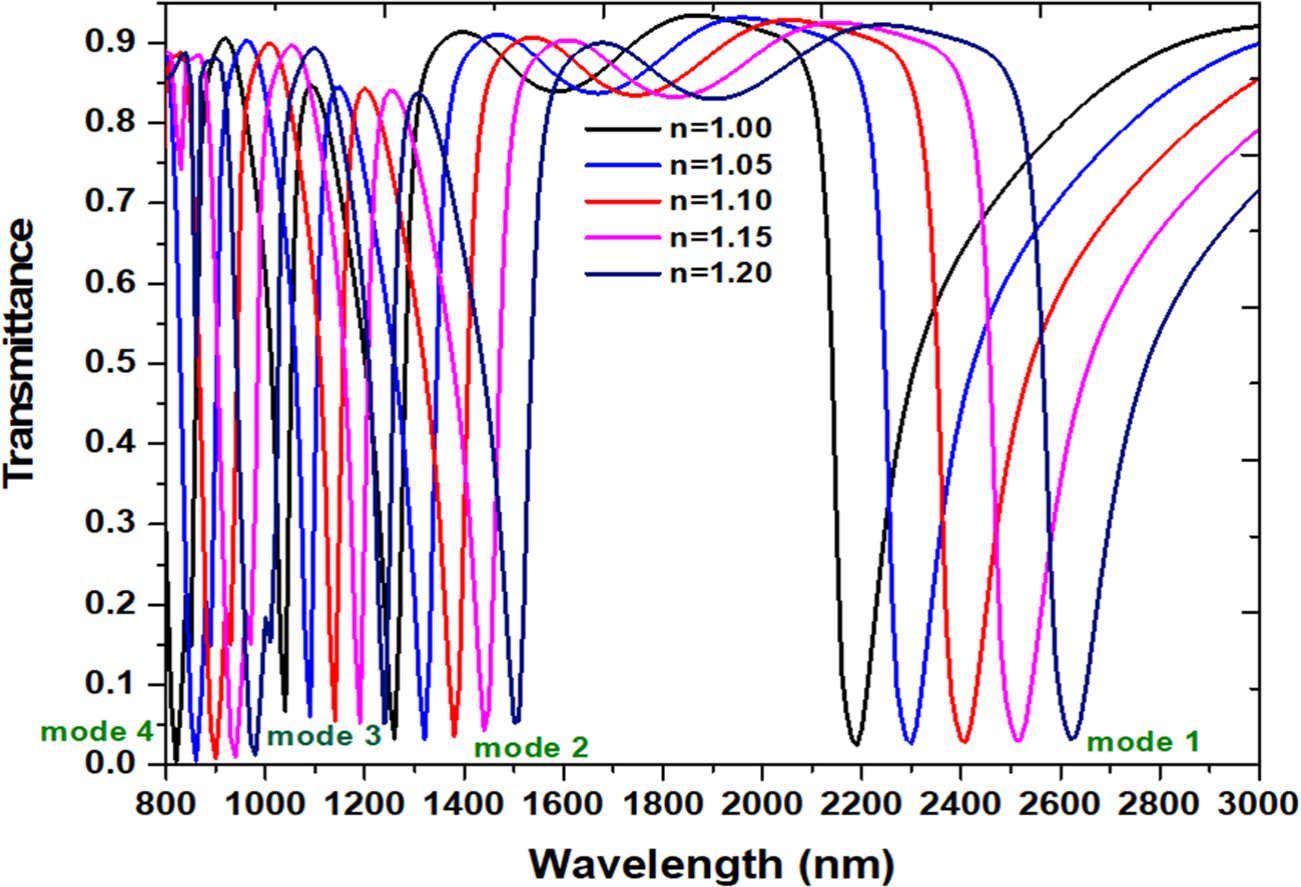
Transmittance spectra of the case 2 structure with the filling media (*n*) of 1.00, 1.05, 1.10, 1.15, and 1.20, respectively. The other parameters keep the same as used in Fig. 2.

The investigated case 2 structure can act as a refractive index sensor and inspect by filling a different detecting medium in a plasmonic waveguide-cavity system. Figure 4 shows the transmittance spectra of the case 2 structure with the filling media, *n*, are 1.00, 1.05, 1.10, 1.15, and 1.20, respectively. The other parameters keep the same as used in Fig. 2. As observed, the transmission dips show a redshift as the increasing refractive index and a linear relationship between the *n*_eff_ and the λ_res_, which reveals a good agreement with Eq. (2). The sensitivity's increment is due to the coupling surface and gap plasmon waves between the bus waveguides and square air ring resonator, which leads to an interaction with the variation in the refractive index^81^.

**Figure 5:**
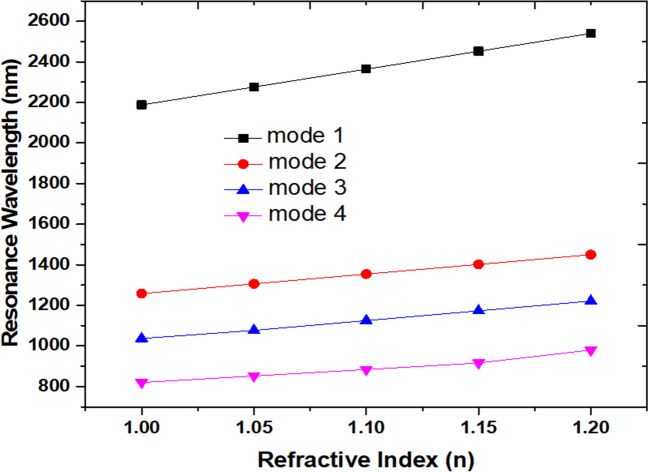
Calculated resonance wavelength (λ_res_) from mode 1 to mode 4 of case 2 structure versus the refractive index.

An excellent refractive index sensor should possess a high sensitivity (S) and acceptable FOM and Q-factor. Figure 5 plots the calculated λ_res_ versus the refractive index of the proposed structure. We summarize the S, FOM, and Q factor and dip strength (ΔT) of case 1 for mode 1 and case 2 from mode 1 to mode 4 in Table 1. Note that the sensitivity values obtained from case 2 from mode 1 to mode 3 can simultaneously achieve above 1000 nm/RIU, which shows excellent sensitivity and acceptable FOM and Q factor. Compared to its typical structure (case 1), the case 2 structure remarkably enhanced the sensitivity by 157%. These values are more noticeable than the previous literature (e.g.,^82–84^) and show multiple modes that can fit the requirement of refractive index sensors in the wavelength of visible and near-infrared.

**Table 1:** The S, FOM, Q factor, and dip strength (ΔT) of case 1 structure for mode 1 and case 2 from modes 1 to 4.

	case 1	Case 2			
	Mode 1	Mode 1	Mode 2	Mode 3	Mode 4
S (nm/RIU)	1400	2200	1200	1000	800
FOM (RIU^-1^)	14.00	22.00	20.00	40.00	22.86
Q factor	13.76	21.88	20.97	41.48	32.80
ΔT	0.705	0.9201	0.8185	0.9015	0.8554

**Figure 6:**
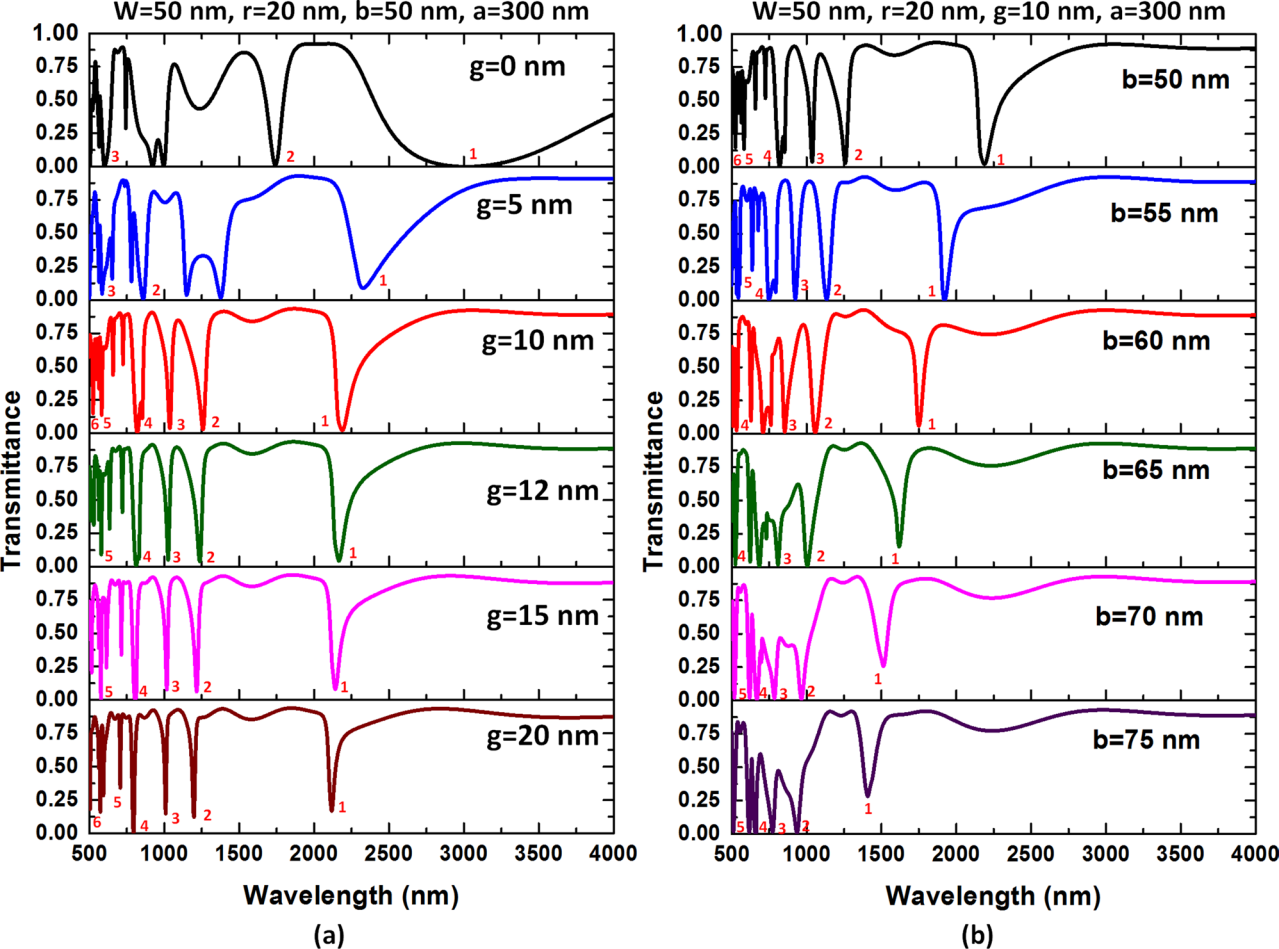
Transmission spectra as a function of (**a**) *g* variation and (**b**) *b* variation of the case 2 structure. The mode number marks in the figures' inset and the structural parameters are at the top of the figures.

**Figure 7:**
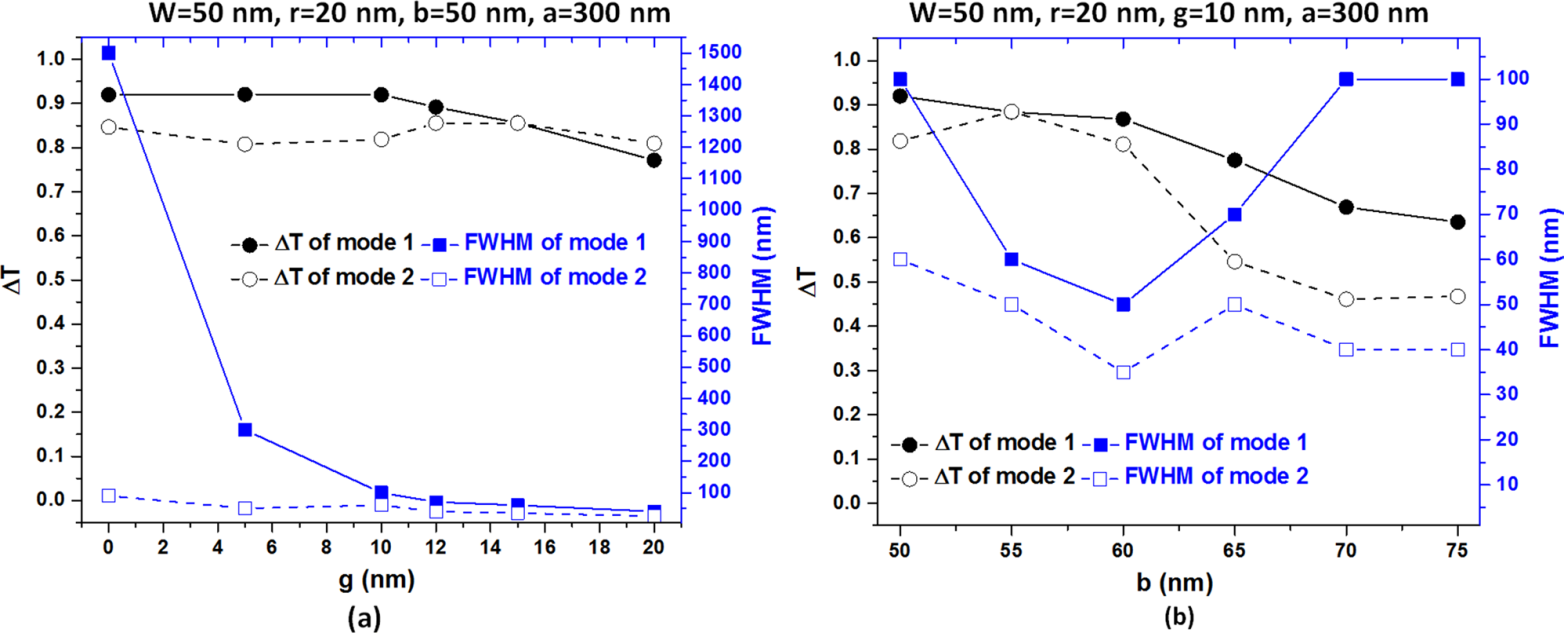
Dip strength (∆T) and FWHM of the proposed type 3 structure in mode 1 and mode 2 for (**a**) varying *g* in the range of (0, 5, 10, 12,15, 20) nm, and (**b**) varying *b* in the range of 50–75 nm in the step of 5 nm. The other structural parameters are at the top of the figures.

The SPPs modes arising from the case 2 structure are due to the coupling effect between bus waveguide and square ring resonator, significantly influenced by the structural size. In case 2 frame, w is fixed at 50 nm to promise that the TM mode can propagate in the bus waveguide. Therefore, in our simulations, we further inspect the other four parameters, i.e., *g, b, a*, and *r*. First, Fig. 6a,b depict the influence of *g* and *b* of the case 2 structure on the transmittance spectrum. Figure 7a,b also illustrate the dip strength (∆T) and FWHM of the proposed case 2 structure in mode 1 and 2 for varying *g* and *b*, respectively. We numbered the available resonance modes in the inset of the figures and listed the structural parameters at the top of the figures. As observed, the transmittance dips blueshifts with the increasing *g* and *b*. The transmittance profiles have different behaviors to the change of *g* and *b* since their different physical nature. In Fig. 6a, the coupling effect between bus waveguide and side-coupled resonator becomes weaker because of the increase of *g.* Note that the transmittance dips show a strong oscillation since the more substantial coupling effect when *g* = 0 nm. Besides, the transmittance dip strength (∆T) and FWHM can significantly reduce with the increase of *g* due to the more negligible coupling effect of a more significant *g.* As seen in Fig. 7a, the values of FWHM of mode 1 decline from 1500 to 40 nm, while those of mode 2 decrease from 90 to 25 nm as *g* varies from 0 to 20 nm. We found that the ∆T of both mode 1 and mode 2 exceeds 0.8122 when *g* is in the range of 0–20 nm. It is evident from Figs. 6b and 7b, the FWHM presents a minimum value around *b* = 60 nm, remaining the dip strength almost constant, ranging in 50–65 nm. According to Figs. 6 and 7, we can choose the availed values of *g* and *b* when *g* is in the range of 10–20 nm, and *b* is in the range of 50–65 nm based on the ∆T, FWHM, transmittance line-shape, and the number of resonance mode.

**Figure 8:**
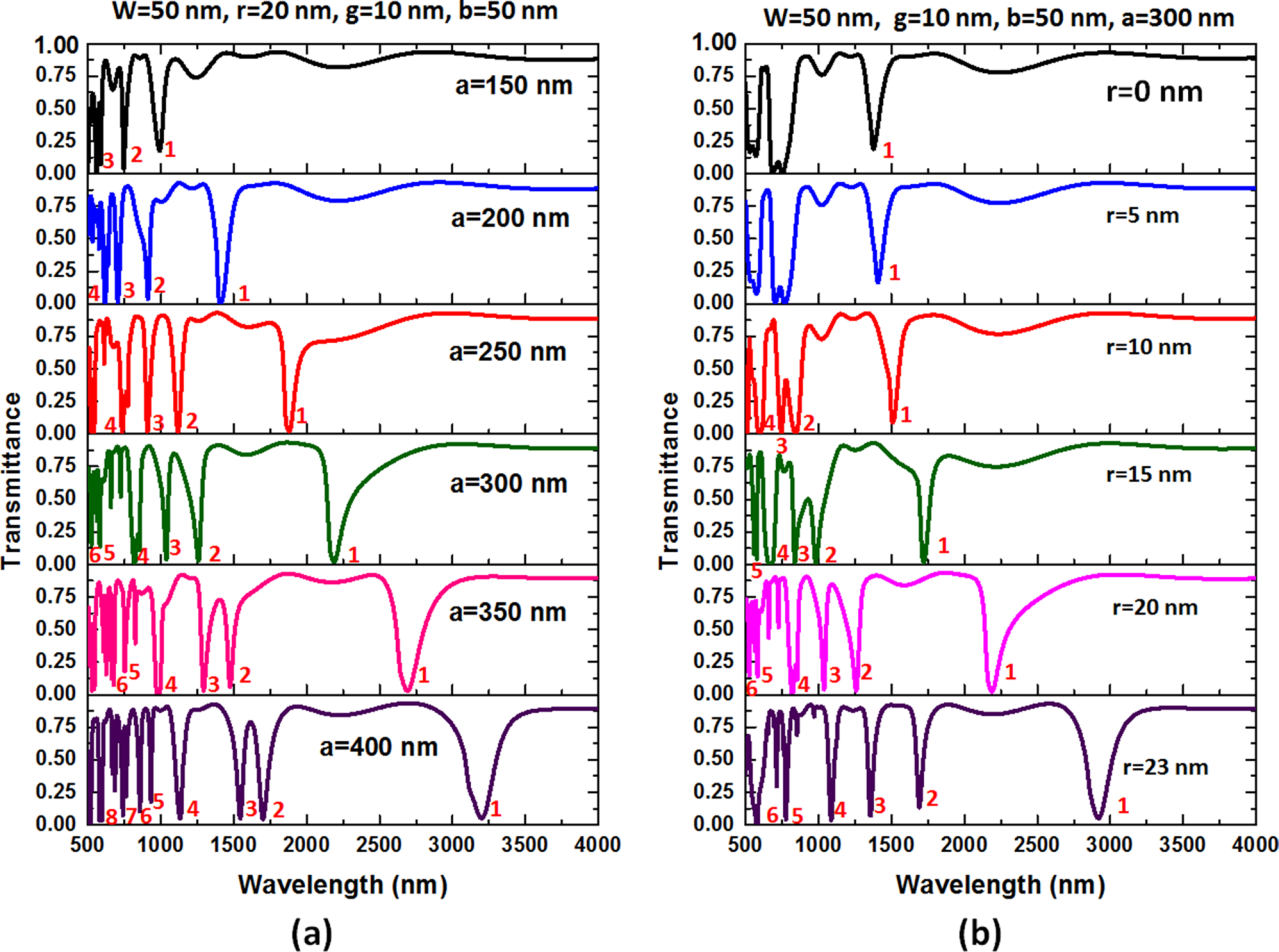
Transmission spectra as a function of (**a**) *a* variation and (**b**) *r* variation of the case 2 structure. The mode numbers label in the figures' inset and the structural parameters are at the top of the figures.

Successively, we show the variation influence of *a* and *r* on the transmittance spectrum in Fig. 8a,b, respectively. As shown, the λres redshifts with increasing of *a* and *r*. Specifically, the shift of λ_res_ by varying *a* and *r* is more sensitive than that of *g* and *b*, e.g., λ_res_ changes from 992 to 3200 nm for mode 1 when *a* varies from 150 to 400 nm, and λ_res_ varies from 1046 to 2948 nm for mode 1 when *r* varies from 0 to 23 nm, correspondingly. It is evident from Fig. 8a, a more significant *a* can provide a longer optical path and results in a more GPR effect among silver nanorods in the square air ring cavity. However, a strong coupling effect causes a shortcoming of broad FWHM since the more indirect coupling strategy could give rise to a more ohmic loss in the resonator influenced by the silver nanorods. Accordingly, it is a bargain between a slender FWHM strength and a more muscular dip strength. When *a* ≥ 300 nm, FWHM increases and declines the FOM. Therefore, we selected *a* = 300 nm as the starting pointing of the further study. The air gap among adjacent silver nanorods can alter the resonance condition and offer a different optical path in the square air ring cavity. In Fig. 8b, the GPR in the resonator gets more substantial with the increasing of *r.* This phenomenon is that the balance of strength of the discrete state in the resonator and the continuum state in the bus waveguide can change by varying *r*, the resonance modes are changed. The FWHM can significantly enlarge with the increasing *r* since the GPR mode is enhanced by increasing *r* in the resonator. As a result, the optimum coupling effect is achieved at *r* = 20 nm based on FWHM and ∆T, as shown in Fig. 8b.

**Figure 9:**
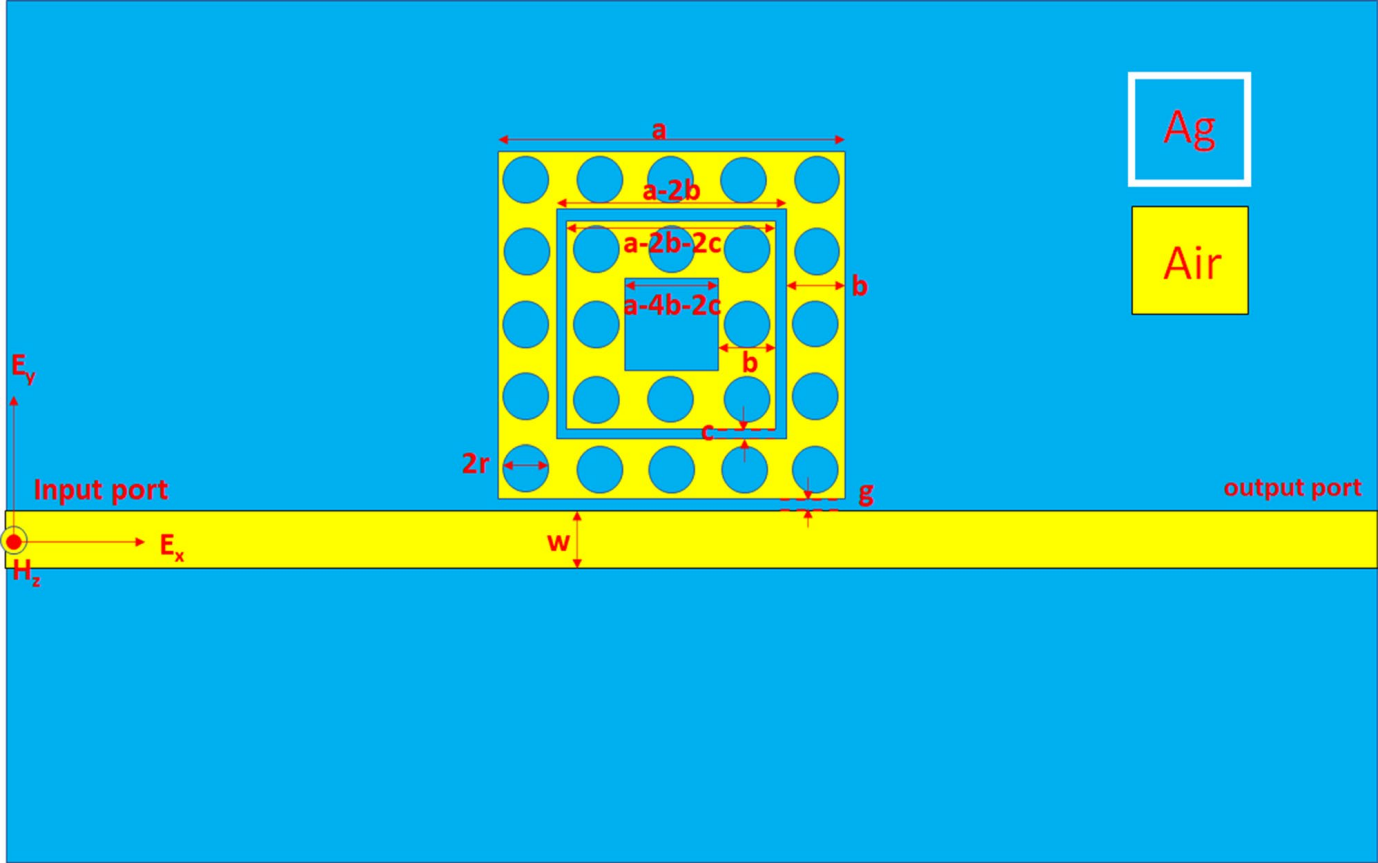
Top view of the proposed case 3 structure, consisting of a MIM bus waveguide side-coupled with two square ring-shaped cavities with sixteen and eight silver nanorods (with radius *r*) uniformly distributed in the first and second rings, respectively. The structural perimeters are in the figure, i.e., the space between the first and second rings is *c*, the gap between the bus waveguide and the first square ring-shaped cavity is *g*, the outer length of the first and second square rings are *a* and *c*, while the internal size of first and second square rings are *a*-2*b* and *a*-2*b-2c*, respectively.

**Figure 10:**
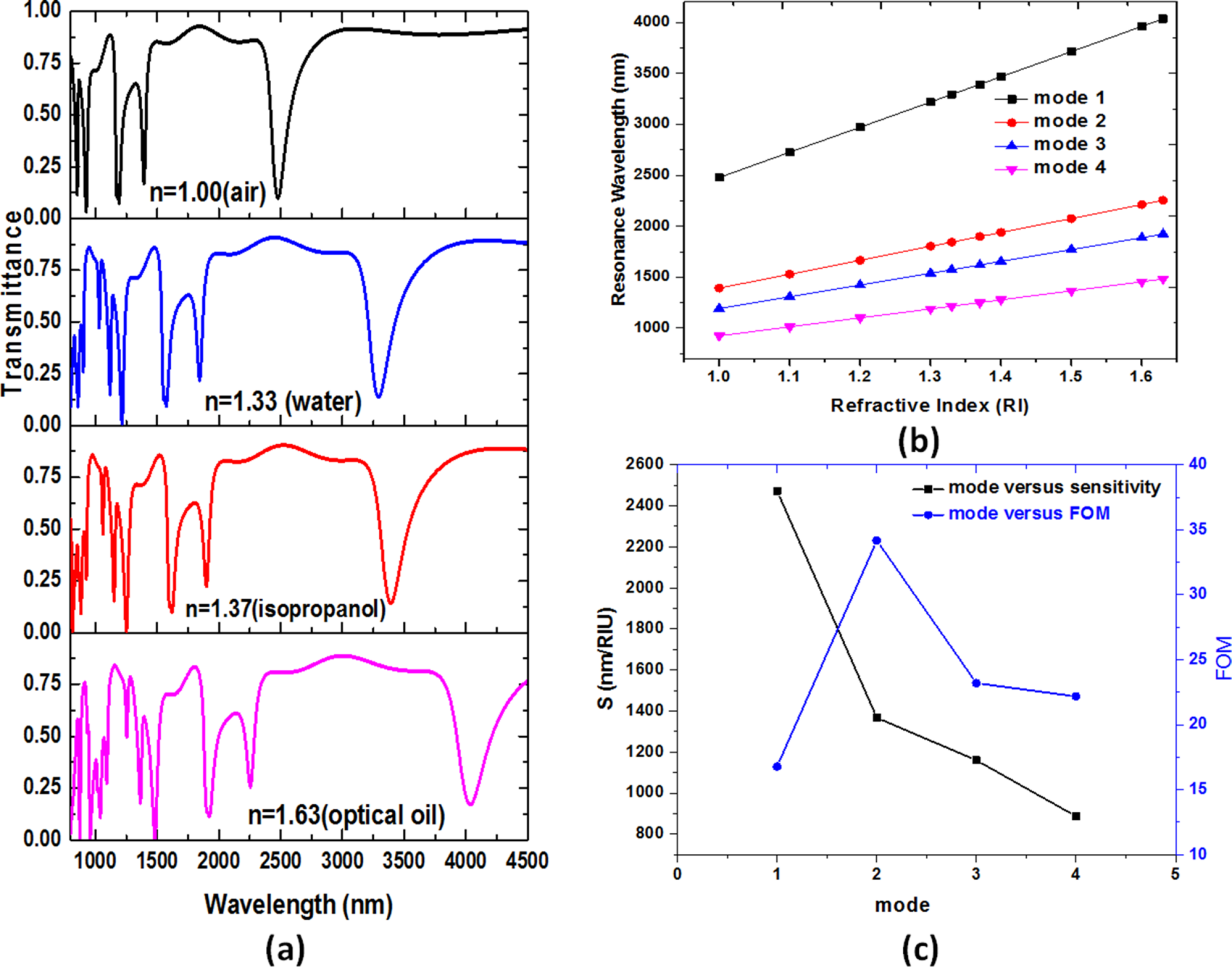
(**a**) Transmittance spectra of case 3 structure at different ambient medium, i.e., water (n = 1.33), isopropanol (n = 1.37) and optical oil (n = 1.63), respectively. (**b**) λ_res_ versus the refractive index value (*n*) from 1.00 to 1.63 of case 3 structure for mode 1 to mode 4. (**c**) S and FOM of case 3 structure from mode 1 to mode 4.

Based on the case 2 structure analysis, we found the coupling effect between the bus waveguide and the side-coupled resonator will enhance by generating more GPR effect inside the coupled resonator. To increase the coupling effect and gap plasmon resonance, we proposed a case 3 structure, i.e., the second air ring is added in the case 2 structure. Figure 9 shows the top view of the proposed case 3 structure, containing a MIM bus waveguide coupled with two square ring-shaped cavities with sixteen and eight silver nanorods (with radius *r*) uniformly distributed and second rings, respectively. The structural perimeters are signified in Fig. 9, i.e., the space between the first and second rings is *c*, the gap between the bus waveguide and the first square ring cavity is *g*, the outer lengths of the first and second square rings are *a* and *a-2b-2c*, while the inner lengths of first and second square rings are *a*-2*b* and *a*-4*b-2c*, respectively. For simplicity, we do not discuss the influence of the *c* value on the plasmonic responses but directly give the optimized value, which is *c* = 10 nm for the case 3 structure. The rest structural parameters, *w, g, r, a, b,* are set as 50 nm, 10 nm, 20 nm, 300 nm, and 50 nm. Figure 10 shows the transmittance spectra of case 3 structure at different ambient medium, i.e., air (n = 1.00), water (n = 1.33), isopropanol (n = 1.37) and optical oil (n = 1.63), respectively. This range of detecting fluids is associated with the biological sample analytes. The fluids are located on the entire structure's upper surface in the refractive index sensing process and consider the infinite thickness. It is evident in Fig. 10a that a remarkable redshift of transmittance dip with the increase of the ambient refractive index. Figure 10b illustrates the λ_res_ versus the refractive index value (*n*) from 1.00 to 1.63 of case 3 structure from mode 1 to mode 4. We found a redshift of λ_res_ with increases *n* in the refractive index range of gas and liquid. Figure 10c depicts the S and FOM of case 3 structure from mode 1 to mode 4 and shows a more massive shift in mode 1 than the other modes.

We summarized the calculated S, FOM, and Q factor of case 3 structure from modes 1 to 4 in Table 2. The sensitivity obtained from modes 1 to 3 of case 3 configuration simultaneously exceeds 1160 nm/RIU, revealing more excellent sensitivity, acceptable FOM, and Q factor than cases 1 and 2 frames. The proposed case 3 structure can significantly improve 177% and 112% sensitivity compared to cases 1 and 2. Furthermore, the combination of the first and second square air rings, including the silver nanorods in case 3 structure, offers a better sensing performance, as shown in Fig. 10b,c and Table 2. In addition, a more quantity of detecting medium can participate in the case 3 structure due to the longer optical path, resulting in more GPR and SPR effects and enhancing the cavity plasmon resonance in the resonator. Therefore, it will significantly benefit the interaction between the testing sample and the proposed plasmonic sensing system.

**Table 2:** The S, FOM, and Q factor of case 3 structure from mode 1 to mode 4.

	Mode 1	Mode 2	Mode 3	Mode 4
S (nm/RIU)	2473	1367	1160	887
FOM (RIU^−1^)	16.78	34.18	23.20	22.18
Q factor	20.18	56.35	27.34	30.05

**Figure 11:**
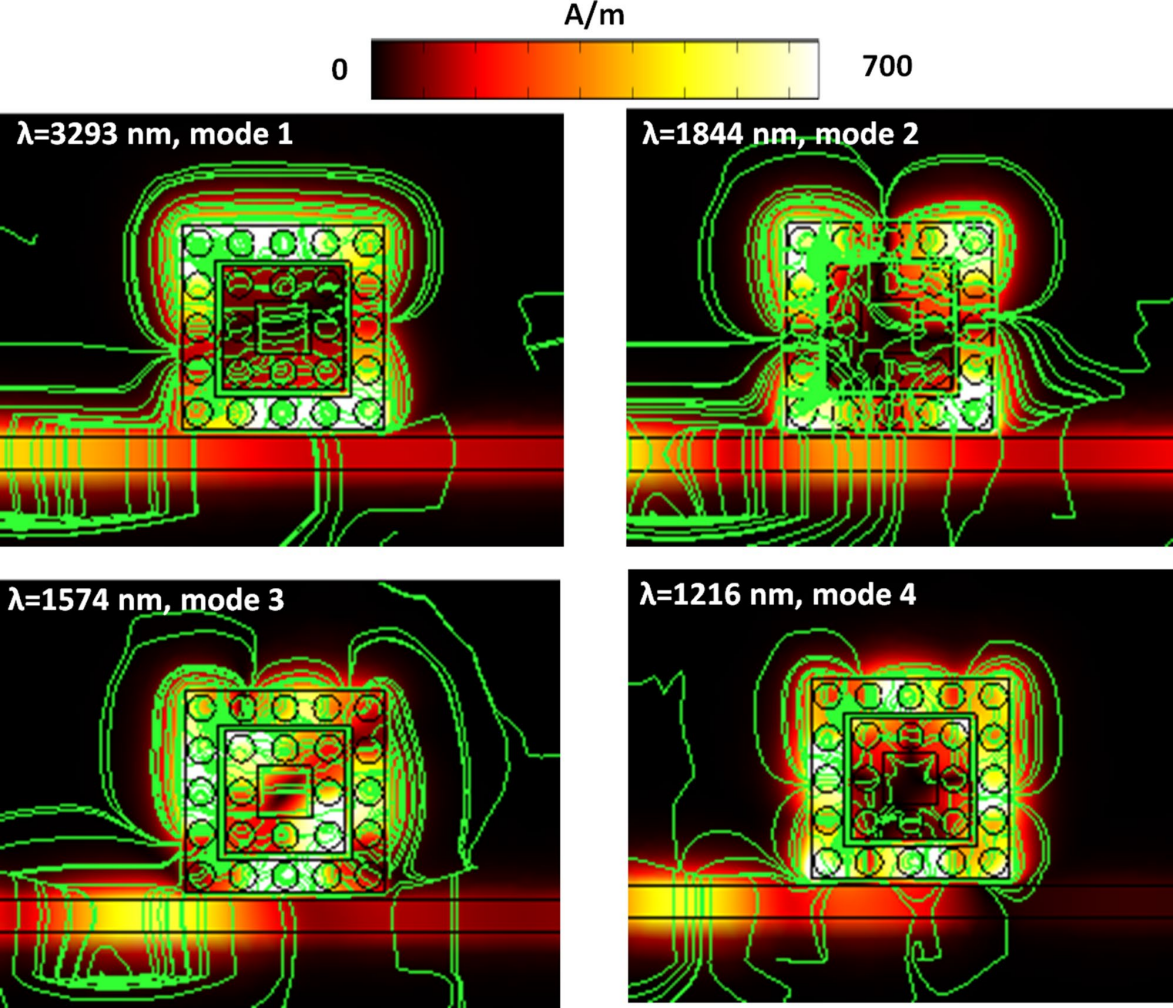
The magnetic field intensity (|H|, including the surface electric force lines (green lines)) of resonant fluids (*n* = 1.33 as an example) around the case 3 structure from modes 1 to 4, respectively.

**Figure 12:**
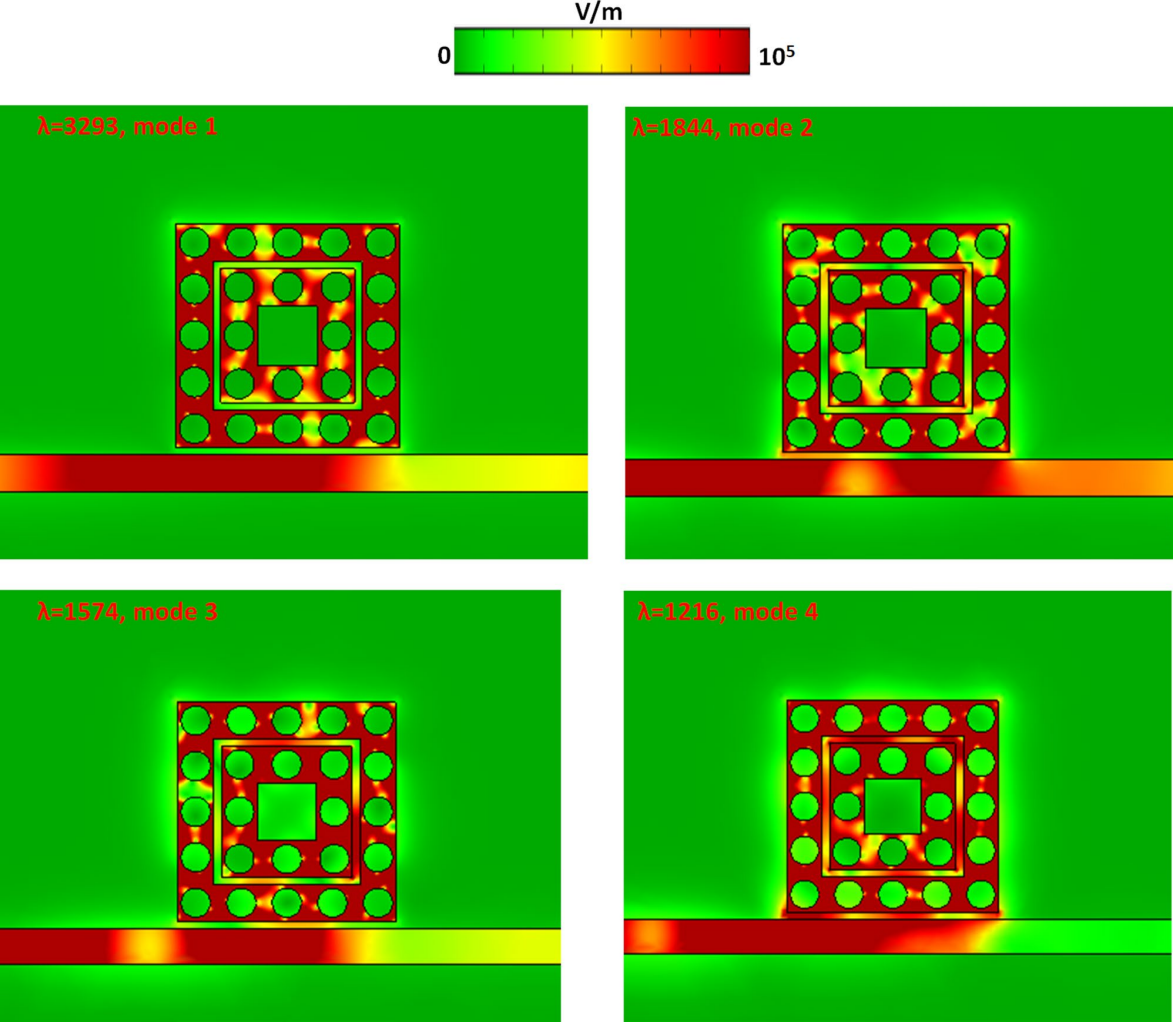
The electric field intensity (|E|) of resonant fluids (*n* = 1.33 as an example) around the case 3 structure from mode 1 to mode 4, respectively.

**Figure 13:**
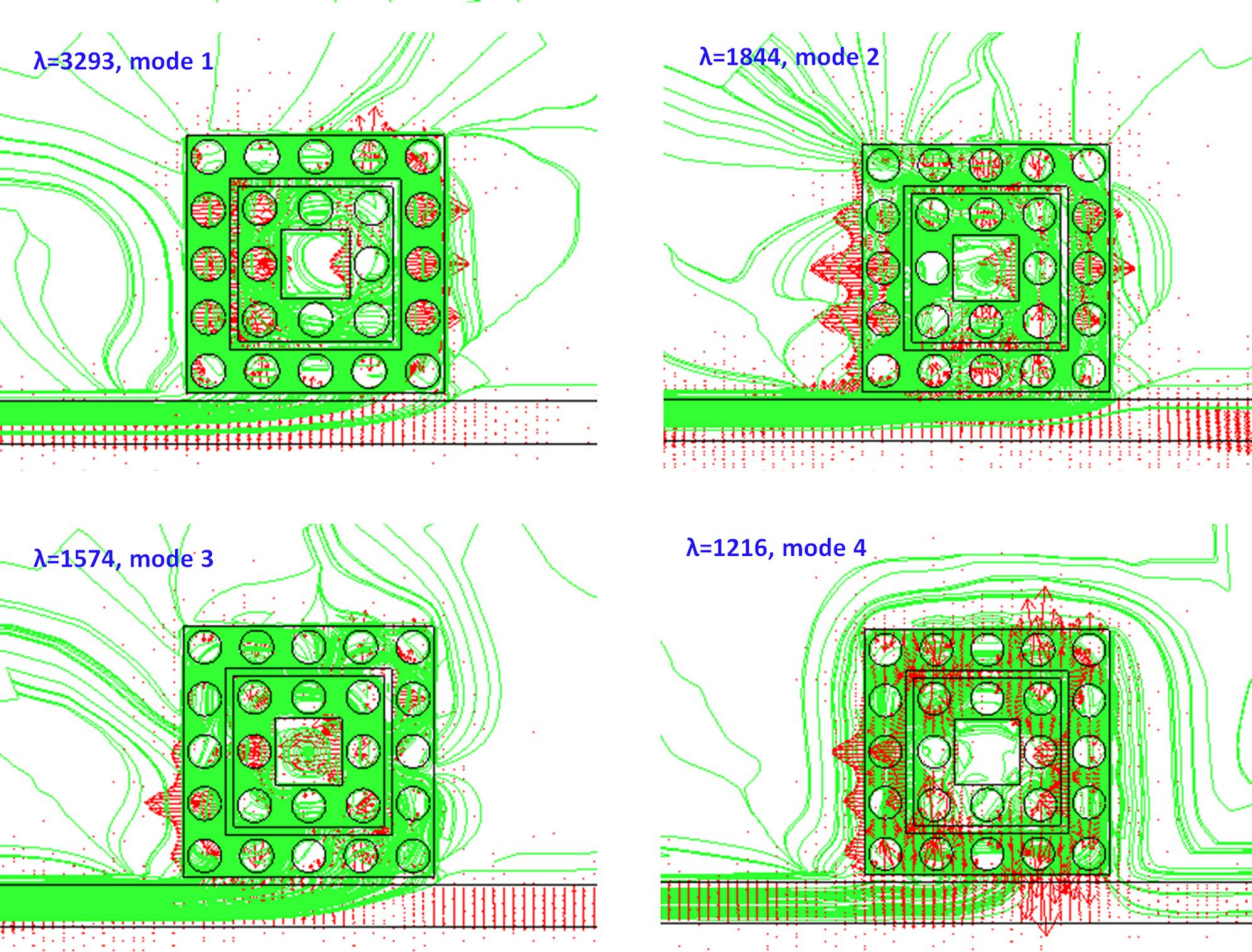
Time average power flow (green lines) with arrows (red arrows) of resonant fluids (*n* = 1.33 as an example) around the case 3 structure from modes 1 to mode 4, respectively.

When the fluid is resonant with the case 3 structure, the transmittance spectrum varies with the refractive index increase, showing an excellent exciton-plasmon coupling and generating a deep bonding mode based on GPR in the resonator. This phenomenon can interpret by the magnetic field intensity (|H|) (including the surface electric force lines (green lines), Fig. 11), electric field intensity (|E|, Fig. 12), and time-average power flow (green lines) with arrows (red arrows) (Fig. 13), respectively. Figures 11, 12, 13 show the occurrence of resonant fluids (e.g., *n* = 1.33 as an example) around the case 3 structure from modes 1 to 4. The cavity resonance in case 3 structure is highly sensitive to the changes in the refractive index. The presence of fluids affects the fluid-field interaction and the spatial distribution of the E-field intensity and the power flows (time average, W/m^2^) across the interface between bus waveguide and resonator of the case 3 structure. Concerning the electric force lines and the power flow arrows, as shown in Figs. 11 and 13, it raises a robust EM Feld localization and enhancement (see Fig. 12) and the power flows in the first and second square air rings. As a result, the effect of the accumulated GPR at λ_res_ meets the Fabry–Pérot resonance condition and forms a strong cavity resonance in the side-coupled resonator. Besides, the EM waves and energy flows show that the enhanced surface plasmon among the gaps of silver nanorods, revealing a broad range of interactions with analytes, thereby demonstrating the potential of the case 3 structure for sensing applications.

The recorded sensitivity and FOM in the case 3 structure are superior to those of previous MIM-cavity systems. We summarize S and FOM comparing this work and other reported similar SPR sensors in Table 3.

**Table 3:** Comparison of the best sensitivity and FOM between this work and some selected published articles.

Reference/year	Cavity Structure	Sensitivity (nm/RIU)	FOM (1/RIU)
^85^/2015	Induced transparency resonator	733	30.50
^86^/2016	Nanowall side-coupled resonator	985	28.20
^84^/2017	Triangle and ellipse-ring resonators	860	31.60
^87^/2018	Tangent-Ring Resonators	880	964.00
^88^/2018	X-shaped resonator cavities	1303	3113 (FOM*)
^89^/2019	Ring shape resonator	636	211.30
^90^/2019	Two side-coupled semi-ring cavities	1405	3.62 × 10^5^ (FOM*)
^91^/2020	Semi-ring shape resonator	1084	57.06
^92^/2020	Double concentric square ring	1270	58.00
^51^/2021	A semi-ring cavity	1550	7358 (FOM*)
^93^/2021	Racetrack ring resonator	1774	61.00
This work	Double square rings with nanorods	2473	34.18

P. S. FOM* = max(|dT(λ)/d*n*(λ)/T(λ) |), where T (λ) is the transmittance, and dT(λ)∕d*n*(λ) is the transmittance change at a fixed wavelength induced by a refractive index change.

The case 3 frame can apply for sensing biological parameters, e.g., glucose concentration, verifying the refractive index. To imitate the real situation in the simulations, we can describe the refractive index of the glucose solution as^94,95^:

5 $$ n_{g} \, = \,0.000{11889}\, \times \,c_{{\text{g}}} \, + \,{1}.{3323}0{545} $$

where *c*_g_ is the glucose concentration (g/L). Equation (5) elucidates the linear relationship between the *n*_g_ and λ_res_.

**Figure 14:**
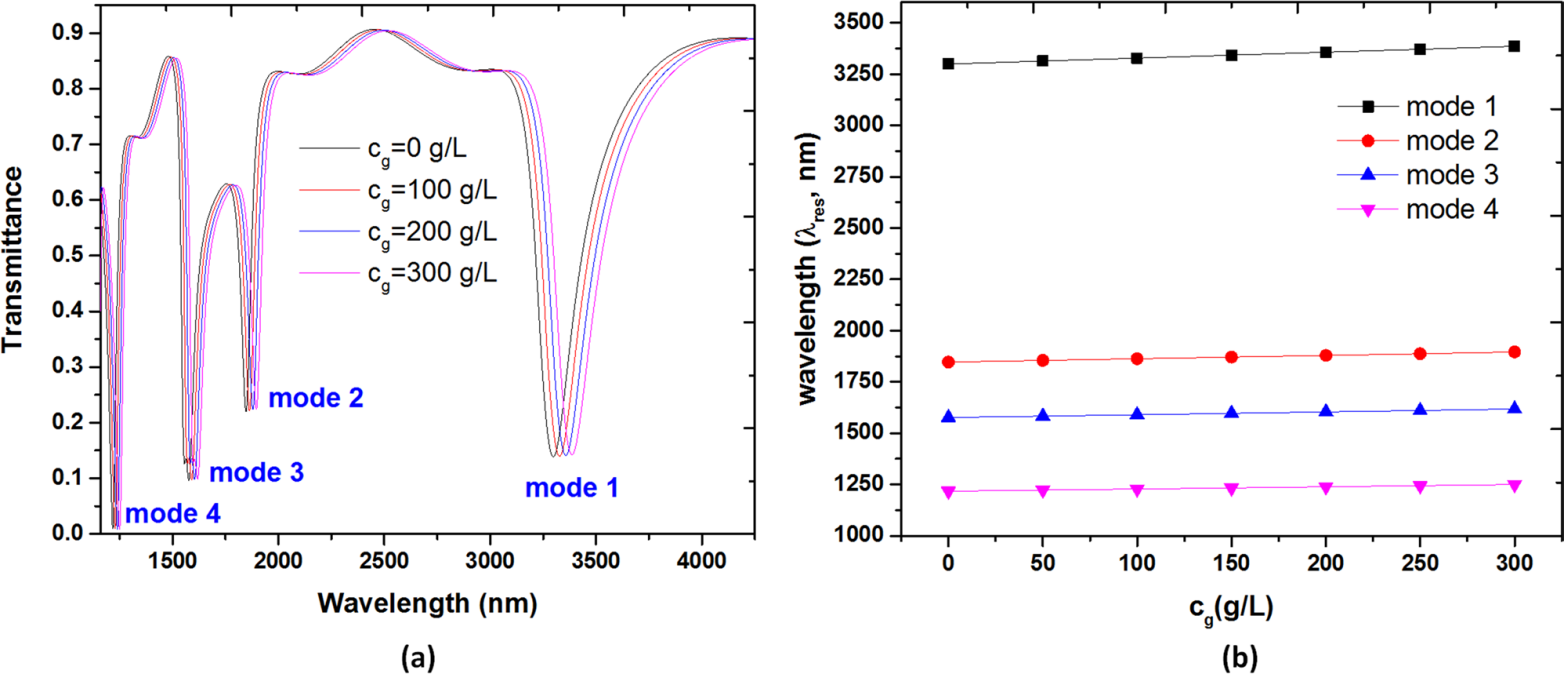
(**a**) Transmittance spectrum of the solution in case 3 structure from mode 1 to mode 4 when the glucose concentration, *c*_g_, varies from 0 g/L, 100 g/L, 200 g/L to 300 g/L, respectively. (**b**) Calculated resonance wavelength (λ_res_) from modes 1 to mode 4 versus the glucose concentration (*c*_g_) range from 0 to 300 g/L. The structural parameters are the same as Fig. 10a.

Figure 14a reveals the transmittance spectrum of the glucose solution in case 3 structure from modes 1 to 4 when the glucose concentration, *c*_g_, varies from 0 g/L, 100 g/L, 200 g/L to 300 g/L, respectively. The structural parameters are the same as Fig. 10a. As observed, the λ_res_ of the transmittance dips exhibits a redshift and all curves show the linear relations with *c*_g_, which is good agreement with Eq. (5). Figure 14b shows the approximately linear relationships between the *c*_g_ and the λ_res_. Thus, the sensitivity of glucose solution sensing is S_g_ = ∆λ/∆c_g_. In these cases, the obtained sensitivity from modes 1 to mode 4 can reach 0.19 nm·L/g, 0.16 nm·L/g, 0.14 nm·L/g and 0.14 nm·L/g from modes 1 to mode 4, respectively.

## Conclusion

This study proposed a plasmonic sensor based on a side-coupled resonator in a MIM-cavity waveguide system for refractive index and biomedical sensor applications. We scrutinized and compared three patterns of resonators, i.e., case 1 (one square ring), case 2 (one square ring with silver nanorods), and case 3 (double square rings with silver nanorods), respectively. The designed structure's EM field distributions and transmittance spectra are studied using 2-D FEM for resonance mode analysis and sensing capability characterization. Results show that the suggested case 3 structure greatly contributes to gap plasmon resonance modes for improving sensing performance. The case 3 structure can significantly improve the sensitivity by 177% compared to its traditional design (i.e., case 1). The best sensitivity and FOM of the sensing devices in mode 1 are 1400 nm/ RIU and 14.00 1/RIU for case 1, 2200 nm/ RIU and 40.00 for case 2, and 2473 nm/ RIU and 34.18 for case 3, respectively, while the maximum recorded Q factor are 13.76, 41.48 and 56.35 for case 1, 2, and 3, respectively. This sensor can widely use in gas and biochemistry since its ease of preparation, excellent sensing performance, and broad working wavelengths with multiple modes.

## Data Availability

The authors declare that all data supporting the findings of this study are available from the corresponding author upon reasonable request.
